# Rpb9-deficient cells are defective in DNA damage response and require histone H3 acetylation for survival

**DOI:** 10.1038/s41598-018-21110-9

**Published:** 2018-02-13

**Authors:** Henel Sein, Kristina Reinmets, Kadri Peil, Kersti Kristjuhan, Signe Värv, Arnold Kristjuhan

**Affiliations:** 1 Department of Cell Biology, Institute of Molecular and Cell Biology, University of Tartu, Riia 23, 51010 Tartu, Estonia; 2 Present Address: Department of Biosciences, Section for Biochemistry and Molecular Biology, University of Oslo, Blindernveien 31, 0371 Oslo, Norway

## Abstract

Rpb9 is a non-essential subunit of RNA polymerase II that is involved in DNA transcription and repair. In budding yeast, deletion of *RPB9* causes several phenotypes such as slow growth and temperature sensitivity. We found that simultaneous mutation of multiple N-terminal lysines within histone H3 was lethal in *rpb9*Δ cells. Our results indicate that hypoacetylation of H3 leads to inefficient repair of DNA double-strand breaks, while activation of the DNA damage checkpoint regulators γH2A and Rad53 is suppressed in Rpb9-deficient cells. Combination of H3 hypoacetylation with the loss of Rpb9 leads to genomic instability, aberrant segregation of chromosomes in mitosis, and eventually to cell death. These results indicate that H3 acetylation becomes essential for efficient DNA repair and cell survival if a DNA damage checkpoint is defective.

## Introduction

To protect genomic integrity, cells must continuously detect and repair DNA damage. Among different types of DNA lesions, double-strand DNA breaks (DSBs) are the most harmful, as they can lead to translocations and deletions of large fragments of chromosomes. To ensure efficient repair of DSBs, cells activate DNA damage checkpoints–mechanisms that halt progression of the cell cycle to provide extra time for DNA repair^1^. Numerous endo- and exogenous factors, including biochemical processes like cellular respiration or gene transcription may lead directly or indirectly to DNA damage. One example of such an endogenous trigger involves collisions between RNA and DNA polymerases that may occur in the S phase of the cell cycle and may in turn give rise to DSBs^2^. In such instances, efficient removal of RNA polymerase from DNA is essential for DSB repair and for continuation of DNA replication^3^.

Transcription of protein-coding genes is conducted by RNA polymerase II (RNAPII), which is comprised of 12 subunits encoded by genes *RPB1* to *RPB12* in yeast. Among these, two subunits–Rpb4 and Rpb9–are non-essential for cell viability and gene transcription. However, their deletion gives rise to several diverse phenotypes such as slow growth, sensitivity to high and low temperatures and to nucleotide-depleting drugs^4–11^. Rpb9 promotes ubiquitylation and degradation of stalled RNAPII in response to UV-induced DNA damage^12^ and is also involved in transcription-coupled repair through its role in regulation of transcription elongation^13–16^. At most RNAPII promoters, selection of the proper transcription initiation start site is altered in the *rpb9* mutant cells^17^. Additionally, Rpb9 is important for maintaining transcriptional fidelity as evidenced by the fact that RNAPII lacking the Rpb9 subunit pauses at obstacles of transcription elongation at a much lower frequency than wild type RNAPII. However, once stopped, the *rpb9*Δ polymerase is inefficient at resuming transcription, as Rpb9 is needed for efficient recruitment of TFIIS–the factor required for activation of nascent transcript cleavage activity of RNAPII and reactivation of the stalled polymerase^18–21^. Although Rpb9 is not essential for cell viability, deletion of *RPB9* is synthetically lethal with disruption of the SAGA complex - the main H3 acetyltransferase in yeast^9,22^, as well as with the Rad6-Bre1 complex^23^ that is required for monoubiquitylation of histone H2B^24,25^. Ubiquitylation of H2B has been implicated both in regulation of RNAPII-dependent transcription and in DNA damage response. It is needed for proper activation of the DNA damage checkpoint, timely initiation of DSB repair, and for recruitment of structure-specific endonucleases to the sites of DNA repair^26–28^. These genetic interactions suggest that chromatin modifications and careful regulation of the DNA damage response become essential for cell viability in the absence of Rpb9.

Acetylation of lysine residues within N-terminal tails of histone proteins is one of the most common chromatin modifications. It weakens histone-DNA and histone-histone interactions, and also serves as a signal for recruitment of several effector proteins. In higher eukaryotes, abnormal patterns of histone acetylation and deregulated expression of chromatin modifiers have been found in various cancers^29–31^. While elevated levels of histone acetylation lead to a more open chromatin in general, some acetylation sites on histone H3 (K14, 23, 56) and histone H4 (K5, 12, 91) have been shown to be important in regulation of DNA repair pathways in particular^32–35^. The precise roles of different histone modifications in this process remain the subject of debate. In fission yeast, acetylation of H3 K14 has been shown to be important for DNA damage checkpoint activation^36^. Specifically, it was found that this modification facilitates DNA repair by directly regulating the compaction of chromatin via recruitment of the chromatin remodelling complex RSC^37^. Another study has revealed that budding yeast strains lacking acetylatable lysines 14 and 23 on histone H3 are sensitive to the DNA-damaging agent methyl methanesulfonate (MMS) and defective in homologous recombination (HR) repair^33^.

To study the role of chromatin modifications in Rpb9-mediated processes, we examined the genetic interactions between Rpb9 and acetylation of histone H3. We found that deletion of Rpb9 was lethal in cells where three or more acetylatable lysine residues were mutated in the H3 N-terminal tail. Our results show that depletion of Rpb9 leads to elevated DNA recombination and impaired activation of the DNA damage checkpoint, while repair of DSBs is inefficient in H3 hypoacetylated cells. When H3 hypoacetylation is combined with depletion of Rpb9, defective DNA damage response and unrepaired DNA lesions lead to genomic instability, aberrant segregation of DNA in mitosis and eventually cell death.

## Results

### H3 acetylation is required for the viability of *rpb9*Δ cells

**Figure 1:**
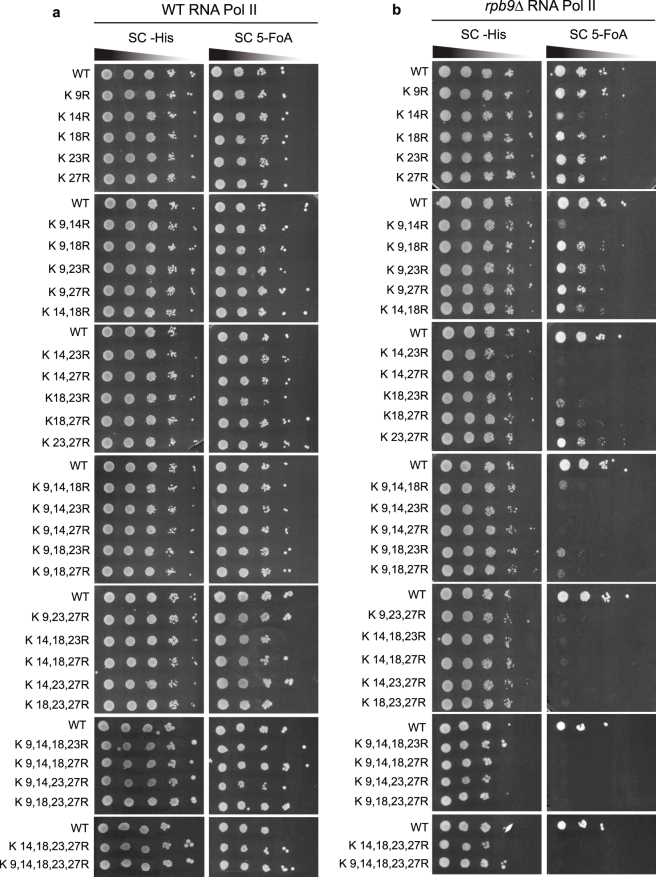
Analysis of genetic interactions between Rpb9 and H3 N-terminal mutations. Cells containing wild type (**a**) or *rpb9*Δ (**b**) RNAPII were transformed with plasmids encoding lysine-to-arginine mutations in histone H3 N-terminal tail. 10-fold serial dilutions of cells were spotted onto synthetic complete (SC) plates lacking histidine, or containing 5-FOA. Plates lacking histidine acted as a control, where strains expressed both wt and mutant versions of H3. On 5-FOA plates, only mutant versions of H3 were expressed. His plates were photographed 3 days and 5-FOA plates 5 days after incubation at 30 °C. Transformation with plasmid encoding wt histones was included on every plate as a control.

RNAPII is directly and indirectly involved in the regulation of DNA transcription, repair and recombination–all processes that require access to DNA in chromatin. Although Rpb9-deficient cells are viable, they display several phenotypes like slow growth and sensitivity to elevated temperatures and genotoxic agents. Genetic interactions have revealed that *RPB9* deletion is synthetically lethal with deletions of the SAGA histone acetyl-transferase complex subunits^9,22^. Based on these observations, we hypothesized that *rpb9*Δ cells might be sensitive to H3 modifications that are crucial for chromatin regulation and genome maintenance. To investigate the role of H3 N-terminal acetylation in *rpb9*Δ cells, we systematically mutated H3 N-terminal lysine residues to arginines to see whether any combination of H3 mutations would affect cell viability. We found that in wild type strain background all H3 mutants were viable and showed no obvious growth defects (Fig. 1a). However, in the *rpb9*Δ strain, several H3 mutations confer lethality (Fig. 1b). While any combination of three or more H3 acetylation site mutations was lethal in the *rpb9*Δ background, some diversity in the phenotypes of H3 double lysine mutants was observed. Specifically, loss of K14 acetylation had the strongest effect on viability of *rpb9*Δ cells, as all non-viable double mutants contained the K14R mutation. However, intact K14 alone could not rescue lethality of *rpb9*Δ cells when three or more other lysine residues were mutated to arginines. This suggests that changes in the overall acetylation levels of the N-terminal tail of H3 may be the prime reason for synthetic lethality with the *RPB9* deletion.

### DNA damage checkpoint activation is impaired in Rpb9-depleted cells

**Figure 2:**
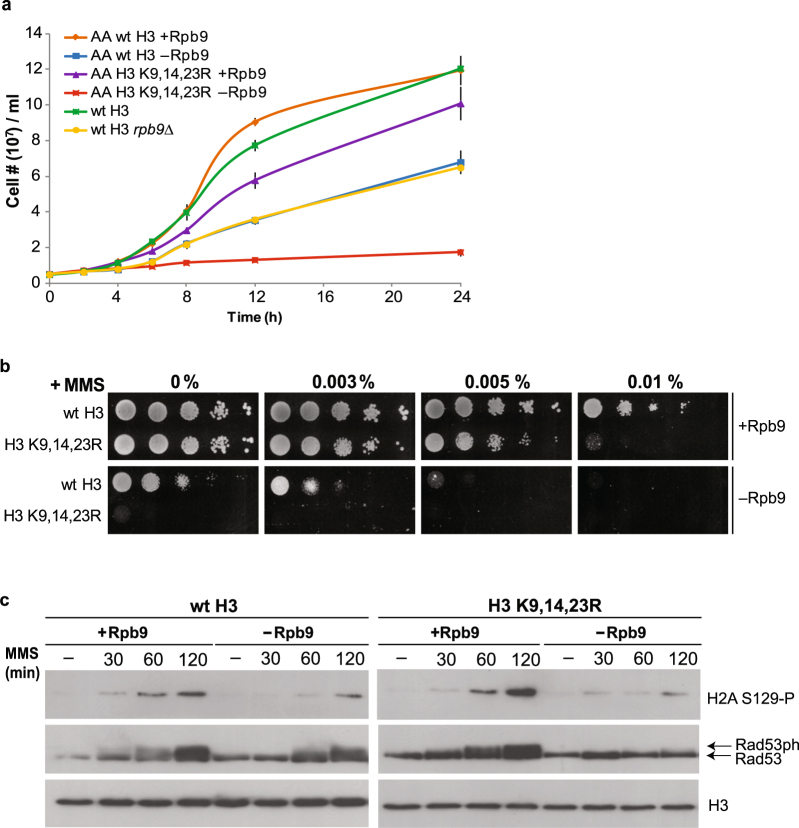
S-phase checkpoint activation is defective in Rpb9-depleted cells. (**a**) Growth curves of Rpb9 anchor-away (AA) strains containing wt H3 or the K9,14,23 R mutant of H3. Rapamycin (−Rpb9) or DMSO (+Rpb9) was added to the cell growth medium at time-point 0, and culture density (cells/ml) was measured at indicated time-points. Wild type (wt H3) and *rpb9*Δ strains were used for reference. (**b**) 10-fold serial dilutions of cells with wt H3 or the H3 K9,14,23 R mutant were spotted onto SC plates containing DMSO (+Rpb9) or rapamycin (−Rpb9) with addition of indicated concentrations of MMS. (**c**) Western blot analysis of H2A and Rad53 phosphorylation in response to MMS treatment in Rpb9-depleted cells. Rpb9 anchor-away strains with wt or K9,14,23 R mutant H3 were incubated with DMSO (+Rpb9) or rapamycin (−Rpb9) for 6 hours before 0.01% MMS was added to the cells and samples were taken at indicated time-points. H3 is shown as a loading control. Full-size blots are shown in the Supplementary Fig. S3.

To investigate the mechanisms leading to lethality of the *rpb9*Δ strain in the H3 hypoacetylation background, we continued our study using the anchor-away method^38^ to remove Rpb9 from the H3 K9,14,23 R strain. This method allows pre-growing cells with intact RNAPII and subsequent removal of Rpb9 from the nucleus via addition of rapamycin to the growth medium, thereby phenocopying *rpb9*Δ cells. Since all combinations of three or more N-terminal lysine mutations of H3 were lethal in the *rpb9*Δ background, we continued our study using the H3 K9,14,23 R mutant as a representative example of H3 hypoacetylation. As *RPB9* deletion causes slow growth in yeast, this phenotype can be used as an indicator of rapamycin-induced loss of Rpb9. When Rpb9 was removed from a strain carrying wt histone H3, cell growth rate decreased to levels comparable with the *rpb9*Δ strain, while depletion of Rpb9 from the H3 K9,14,23 R strain arrested cell growth entirely (Fig. 2a). These results confirmed that the anchor-away depletion of Rpb9 was efficient in our model system and was suitable for further studies of Rpb9-dependent survival of H3 K9,14,23 R cells. We additionally confirmed the efficiency of Rpb9 depletion by a spotting assay on rapamycin-containing media, where it was lethal in the H3 K9,14,23 R background (Supplementary Fig. S1).

As Rpb9 is involved in DNA repair, we tested whether Rpb9-depleted, or H3 K9,14,23 R mutant cells can properly respond to DNA damage induced by MMS. While H3 K9,14,23 R mutation caused relatively mild MMS-sensitivity, the Rpb9-depleted cells were highly sensitive to long-term exposure to MMS (Fig. 2b). We confirmed that this result was not restricted to MMS treatment, as DSB induction with ionizing radiation or camptothecin caused identical phenotypes (Supplementary Fig. S2). Given that both Rpb9-depleted and H3 K9,14,23 R cells were sensitive to MMS, we hypothesized that these mutations may affect different steps in DNA repair pathway that can be tolerated separately, but become synthetically lethal in an Rpb9-deficient H3 K9,14,23 R strain.

In eukaryotic cells, genomic stability is maintained through careful coordination of DNA damage repair and cell cycle control. DNA damage checkpoints become activated to arrest the cell cycle, thereby allowing additional time for repair of DNA lesions. To test whether Rpb9-depleted cells can properly activate DNA damage checkpoints, we followed the kinetics of H2A and Rad53 phosphorylation in response to MMS treatment of cells. Phosphorylation of H2A Ser129 (γH2A) is one of the earliest checkpoint activating events that leads to Rad9-mediated recruitment and autophosphorylation of Rad53, and subsequent phosphorylation of multiple targets by Rad53^39–41^. We found that both wild type and H3 K9,14,23 R cells responded quickly to MMS, while DNA damage checkpoint activation was impaired in Rpb9-deficient cells (Fig. 2c). This indicates that activation of the γH2A-Rad9-Rad53 pathway is impaired in the absence of Rpb9 and that cells lacking this RNAPII subunit cannot adequately respond to DNA damage. Impaired activation of the DNA damage checkpoint in the Rpb9-depleted strain suggests that these cells may progress through the cell cycle with unrepaired DNA. Under normal growth conditions, this may merely lead to a decrease in the overall growth rate of the strain as only a subpopulation of cells acquires lethal amounts of DNA damage. However, if extensive DNA damage is induced throughout the entire cell population by treatment with genotoxic agents, none of the cells can survive without proper DNA damage checkpoint activation.

### Depletion of Rpb9 leads to DNA repair by homologous recombination

**Figure 3:**
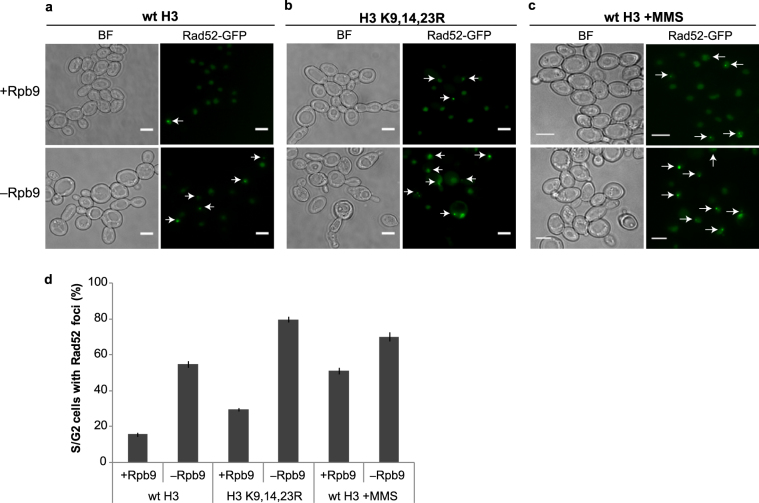
Homologous recombination foci accumulate in Rpb9 depleted cells. Formation of Rad52-foci in response to 6-hour depletion of Rpb9 in cells with wt H3 (**a**), or H3 K9,14,23 R mutant (**b**). Additional DNA damage was induced in wt H3 cells with MMS (**c**). Scale bar 5 µm. (**d**) Quantification of S/G2 phase cells with Rad52-foci determined from three separate experiments.

Although DNA damage checkpoint activation was impaired in Rpb9-deficient cells, this did not explain the synthetic lethality of Rpb9 depletion and H3 hypoacetylation. With no exogenous DNA damage, the checkpoint functionality is not required for cell survival, as many checkpoint-deficient strains, such as *mec1*Δ and *rad53*Δ hypomorphs, and *rad9*Δ, are viable^42–44^. This suggests that in addition to aberrant checkpoint activation, Rpb9-deficiency actually induces genomic instability. To test whether depletion of Rpb9 leads to increased DNA damage, we determined the relative amount of cells with HR foci upon depletion of Rpb9 with and without exogenous induction of DNA damage by MMS. We used GFP-tagged Rad52 protein to reveal recombination sites in cells. During the S phase, Rad52 accumulates at nuclear foci that are indicative of active DNA repair by HR^45^. With no exogenous induction of DNA damage, Rad52 foci appeared in cells 6 hours after Rpb9 depletion (Fig. 3a). Importantly, Rpb9-depletion resulted in approximately the same amount of cells with Rad52 foci as did MMS treatment of wild type cells (Fig. 3c and d), highlighting the severity of DNA damage in the absence of Rpb9. When Rpb9-depleted strain was treated with MMS, the proportion of cells with Rad52 foci increased even further, reaching approximately 75% of the cell population (Fig. 3c and d). Interestingly, the elevated steady-state level of HR in the Rpb9-depleted strain coincides with delayed activation of the DNA damage checkpoint in these cells (Fig. 2c), suggesting that the checkpoint signalling might be saturated by the high background level of DNA repair. Moderate increases in numbers of Rad52 foci were also observed in H3 K9,14,23 R cells. When Rpb9 was depleted in this strain, Rad52 foci were detected in nearly 80% of cells (Fig. 3b and d). These results indicate that H3 K9,14,23 R and depletion of Rpb9 have a cumulative effect on induction of HR, suggesting that they act in different pathways of DNA repair.

### H3 K9,14,23 R cells are ineffective in DSB repair and require DNA damage checkpoint activation for survival

**Figure 4:**
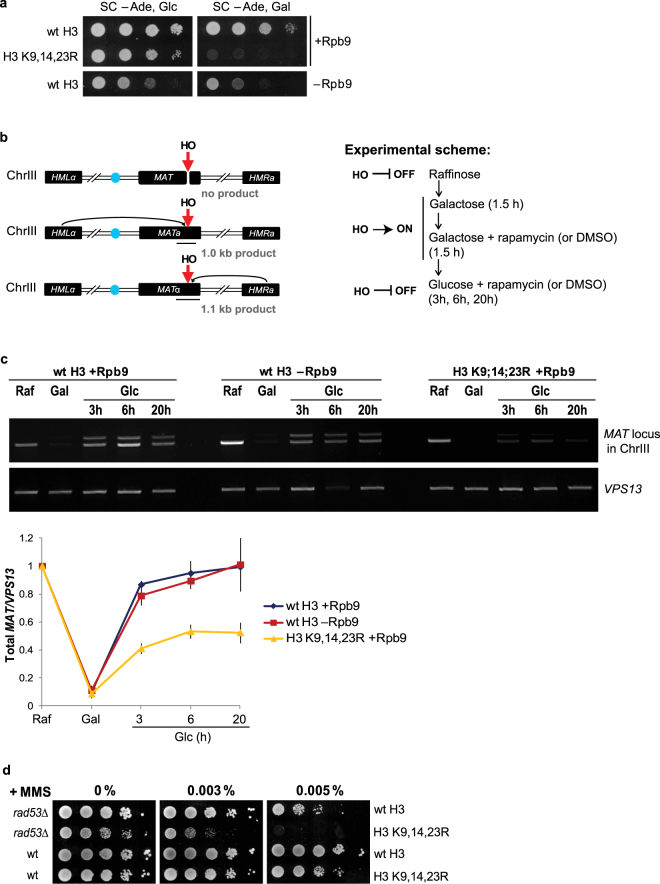
DSB repair is impaired in H3 K9,14,23 R cells. (**a**) Strains with wt H3 or H3 K9,14,23 R were transformed with GAL-HO-pRS412 plasmid and 10-fold serial dilutions of cells were spotted onto SC media lacking adenine and containing either glucose (−Ade, Glc) or galactose (−Ade, Gal). Plates were photographed after 3 days of growth at 30 °C. (**b**) Outline of the DSB repair assay. Schematic representation of the mating type (*MAT*) locus and silent mating loci (*HML*α and *HMR*a) in chromosome III is shown on the left. The site of HO cutting is indicated by red arrow, the centromere of the ChrIII is indicated as a blue circle and the approximate locations of PCR products shown on panel C are indicated as black thin lines under the *MAT* loci. The experimental scheme is shown on the right. The HO endonuclease was expressed under the control of a galactose-inducible promoter. Cells were pre-grown in raffinose-containing media where the expression of HO is off. HO was induced by galactose for 3 hours and repressed by shifting cells to glucose-containing media. The intactness of the *MAT* locus was assayed by PCR that spanned the HO cut site and generated a 1.1 kb product in *MAT*α cells or 1.0 kb product in *MAT*a cells. For more detailed description of the assay see Supplementary Fig. S4. (**c**) Top: Agarose gel electrophoresis analysis of HO cutting and repair of the *MAT* locus. PCR products were obtained from cells before HO induction (Raf), 3 hours after HO induction (Gal) and 3, 6 and 20 hours after repression of HO (Glc). Note that during the repair, either *MATa* or *MATα* genes can be copied into *MAT* locus. PCR product of the *VPS13* locus was used as an internal control. Bottom: quantification of *MAT* locus repair from at least three independent replicates, the error bars indicate standard error. *MAT*a/α signals were quantified, normalized to the *VPS13* signal and plotted relative to raffinose time points. Full-size blots are shown in the Supplementary Fig. S5. (**d**) 10-fold serial dilutions of *rad53*Δ cells combined with wt H3 or H3 K9,14,23 R were spotted onto MMS-containing plates.

Accumulation of DNA damage upon depletion of Rpb9 suggests that survival of these cells depends largely on the efficiency of DNA repair, and that any factor diminishing its effectiveness may become lethal. Given the sensitivity of the H3 K9,14,23 R strain to MMS (Fig. 2b), we next evaluated the efficiency of DSB repair in these cells. We transformed the strains with plasmid expressing the HO endonuclease under the control of a galactose-inducible promoter. The HO endonuclease introduces a single DSB at its recognition site in the *MAT* locus that is repaired primarily by HR using the silent *HMR* or *HML* loci as donor sequences^46^. Strains that are defective in repair of HO-induced DSB are not able to grow in the presence of continuously expressed HO endonuclease. Both wt H3 and H3 K9,14,23 R cells were able to grow on glucose-containing media, where expression of the nuclease was repressed. In contrast, when the HO nuclease was continuously activated on galactose-containing media, only cells with wt H3 were able to grow, indicating that repair of the HO-induced DSB was ineffective in the H3 K9,14,23 R strain (Fig. 4a). To estimate the efficiency of DSB repair in H3 K9,14,23 R cells, we followed the recovery of the *MAT* locus after shut-down of HO expression in wt H3 and H3 K9,14,23 R strains (Fig. 4b; detailed description of the assay is presented in the Supplementary Fig. S4). While the *MAT* locus was fully restored in cells with wt H3, it was repaired approximately in half of the H3 K9,14,23 R cells. Notably, depletion of Rpb9 did not influence the efficiency of DSB repair at the *MAT* locus (Fig. 4c). These results confirm that H3 acetylation is essential for efficient DSB repair and indicate that H3 hypoacetylation is lethal in the absence of Rpb9-mediated DNA damage checkpoint activation.

These results also suggest that H3 acetylation may become critical for the survival if cells fail to properly activate DNA damage checkpoint. To test this hypothesis, we introduced H3 K9,14,23 R mutation into the checkpoint-deficient *rad53*Δ background. This strain was viable, although it grew slower than *rad53*Δ with wt H3 and was very sensitive to DNA damaging agents (Fig. 4d and Supplementary Fig. S2), confirming the importance of H3 acetylation in cells with a compromised DNA damage checkpoint. However, the H3 K9,14,23 R mutation causes a more severe phenotype in *rpb9*Δ cells (Fig. 2b) than in those lacking Rad53, suggesting that the elevated level of DNA lesions in *rpb9*Δ strain becomes detrimental to cell survival.

### Rpb9-depleted H3 K9,14,23 R cells exhibit abnormal morphology and go through lethal mitosis

**Figure 5:**
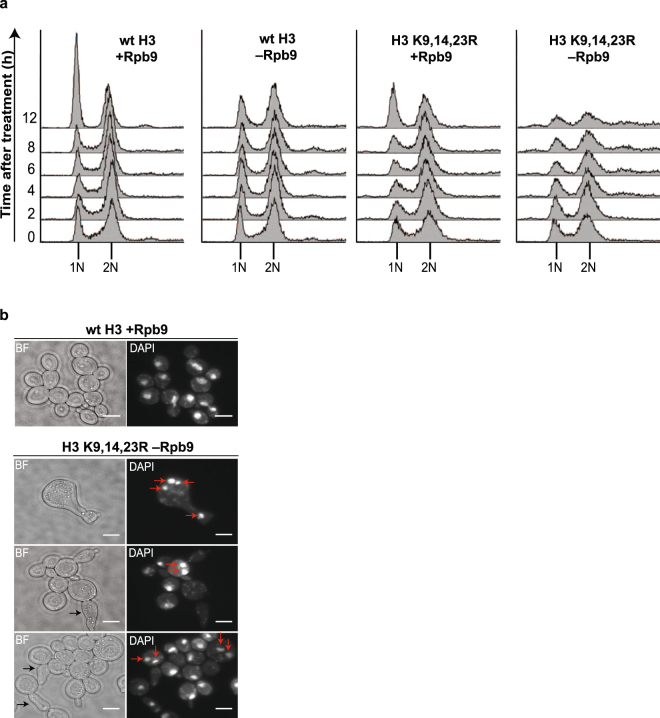
Cell cycle and morphology analysis of Rpb9-depleted H3 K9,14,23 R cells. (**a**) Cell cycle analysis of Rpb9 anchor-away strains with wt or K9,14,23 R mutant H3. Rapamycin was added to growth media of Rpb9 anchor-away strains at time-point 0. Samples were collected at indicated time-points and DNA content of cells was analysed by flow cytometry. (**b**) Rpb9 was depleted from H3 K9,14,23 R cells for 4 hours, after which the cells were fixed and stained with DAPI. Red arrows show aberrations in DNA segregation and black arrows point to abnormally oblong and elongated buds. Scale bar 5 µm, BF–bright field.

To further examine the reasons behind the lethality in Rpb9-deficient H3 K9,14,23 R cells, we analysed cell cycle distribution and DNA content in the H3 K9,14,23 R strain upon depletion of Rpb9. Remarkably, soon after removal of Rpb9, DNA content in this strain becomes heterogeneous, with some cells containing less DNA than in the normal G1 cells and others with abnormally high DNA content indicative of irregular DNA ploidy (Fig. 5a). This suggests that in the absence of Rpb9, H3 K9,14,23 R cells go through mitosis with unrepaired DNA, leading to unequal distribution of the genome between the daughter cells. To confirm this, we studied cell morphology and DNA distribution in H3 K9,14,23 R cells upon Rpb9 depletion. Compared to wild type cells, most of the Rpb9-depleted H3 K9,14,23 R cells displayed a swollen cell phenotype. Additionally, there was a large increase in the number of cells with aberrantly elongated bud morphologies and defects in chromosome segregation (Fig. 5b). Thus, we conclude that collectively inefficient DNA repair and impaired checkpoint signalling lead to unequal mitotic distribution of genomic DNA in Rpb9-deficient H3 K9,14,23 R cells, resulting in aneuploidy and ultimately cell death.

## Discussion

The role of Rpb9 is not unambiguously clear as the cells lacking this protein display a variety of defects, including impaired efficiency of RNAPII elongation and transcription-coupled repair^6,14,15,18,19^, transcription start site selection^17^, impaired degradation of RNAPII in response to UV-induced DNA damage^12^, proteotoxic stress and shortened life span^47^. In order to identify the histone modifications that are critical for viability under stress conditions, we screened a panel of H3 N-terminal lysine-to-arginine mutants in yeast cells lacking the Rpb9 protein, a non-essential subunit of RNAPII. While mutations of acetylatable H3 N-terminal lysines had no detectable effect on survival or growth in cells with wt RNAPII, simultaneous mutation of several N-terminal H3 lysines was lethal in the *rpb9*Δ background (Fig. 1). In particular, loss of K14 acetylation had the strongest effect, as this was the single common site in all lethal double-lysine mutants. However, any combination of three or more mutated lysines in H3 became lethal in the *rpb9*Δ background, suggesting that the overall hypoacetylation of the H3 N-terminal tail was the primary cause for inviability of the cells. Synthetic lethality of hypoacetylated H3 together with loss of Rpb9 is in concordance with a previous finding that *RPB9* deletion is not tolerated in strains lacking the main H3 acetyltransferase Gcn5 or other components of SAGA complex^22^. In this respect, mutation of K14 is indeed expected to have the strongest influence on the acetylation state of H3, as this residue is the preferred target of the SAGA^48,49^. Moreover, acetylated K14 interacts with the bromodomain of Gcn5, and this interaction strongly influences acetylation of other H3 N-terminal lysine residues by the enzyme^50,51^.

We found that removal of Rpb9 induced appearance of active homologous recombination centres. This induction was very prominent, comparable with treatment of the cells with the genotoxic agent MMS. Rad52 foci characteristic of HR emerged in response to Rpb9-depletion, indicating that DNA damage was detected and actively repaired in these cells (Fig. 3). The initial origin of DNA damage is unclear, but it is reasonable to assume that collisions between RNA and DNA polymerases and concurrent collapse of replication fork may induce DSBs and elevated DNA recombination activity in these sites^2^. Several mechanisms have evolved to minimize these collisions^52^, but in the rare cases they do occur, replication can resume only after the RNA polymerase is removed from the site. In this respect, it is important to note that degradation of stalled RNAPII is inefficient in *rpb9*Δ cells^12^, suggesting that proper resolution of transcription-replication collisions may be hampered in the absence of Rpb9, which in turn may lead to elevated levels of DSBs. This is also supported by the fact that high levels of DNA recombination and impairment of replication fork progression were observed in the *rpb9*Δ strain^3^.

We also found that cellular response to DNA damage was compromised in the absence of Rpb9. The principal checkpoint signals of DSBs–phosphorylations of H2A and Rad53–were delayed in Rpb9-deficient cells (Fig. 2c). This finding was unexpected, particularly in light of the fact that DNA damage was actively repaired by HR in the absence of Rpb9. We propose that the excessive DNA damage in these cells exhausts the checkpoint signalling machinery, depleting some essential component(s) of the pathway. For example, the ssDNA binding protein RPA may become limiting in conditions of replication stress, and this may hamper the efficiency of DNA damage checkpoint activation^53,54^. Nevertheless, Rpb9-deficient cells are viable with wt H3, indicating that defective DNA damage checkpoint activation can be compensated by H3 acetylation. This suggests that in the presence of extensive DNA damage, acetylation of histones is essential, as even a minor decline in DNA repair efficiency can be detrimental to cell viability. Indeed, our results confirm that repair of DSBs is inefficient in the H3 K9,14,23 R strain (Fig. 4c), indicating that H3 acetylation may become critical for cell survival in the presence of elevated levels of DSBs. Alternatively, H3 acetylation can be required for viability of DNA damage checkpoint-deficient cells in general. To test that, we introduced the H3 K9,14,23 R mutation into the checkpoint-defective *rad53*Δ strain. These cells remained viable; however, they were very sensitive to DNA damage, confirming that H3 acetylation becomes crucial for cell survival when DNA damage checkpoint is not functional (Fig. 4d and Supplementary Fig. S2).

We found that removal of Rpb9 form H3 K9,14,23 R cells resulted in unequal distribution of DNA between daughter cells as well as large variations in cell morphology (Fig. 5). Analysis of the cell cycle profile revealed aberrant DNA content in most cells and no clearly visible G1 and G2 subpopulations. Collectively these results suggest that Rpb9-deficient H3 K9,14,23 R cells enter mitosis with unrepaired DNA, leading to mitotic catastrophe and cell death. In conclusion, our findings demonstrate that despite accumulation of active HR centres in Rpb9-deficient cells, DNA damage checkpoint is not properly activated in the absence of Rpb9. In these conditions, cell survival depends on efficient DNA repair, which is supported by H3 acetylation.

## Methods

### Yeast strains, plasmids and antibodies

All *Saccharomyces cerevisiae* strains were derived from W303 background and are listed in Supplementary Table S1. Strains AKY796 (wt RNAPII) and AKY1037 (*rpb9*Δ) were used in plasmid shuffling assays. These strains express wild type copies of *HHT2* and *HHF2* from a *URA3*-based plasmid (YCp50:HHT2-HHF2) as a sole source for histones H3 and H4. Histone H3 point mutations were made in *HIS3*-based plasmids (pRS413-H3H4-3F12), transformed into AKY796 and AKY1037, and counter-selected on 5-FOA plates to obtain strains with H3 mutations. All histone expression plasmids are listed in Supplementary Table S2. To generate Rpb9 anchor-away strains, the *RPB9* locus was replaced with *rpb9-FRB-hphMX* expression cassette in strain HHY168 (Euroscarf)^38^. In Rpb9 anchor-away strains AKY1162 and AKY1190, *HIS3*-based plasmid with wild type H3 or H3 K9,14,23 R mutant was introduced as a sole source for H3 and H4 genes. The *rad53*Δ strain was constructed by first replacing the *SML1* gene with kanMX6 (AKY1438) and then *RAD53* was replaced with *URA3* to obtain AKY1459 (*RAD53* deletion is viable only in *sml1*Δ background). yEGFP was fused to C-terminus of Rad52 in its native locus in AKY1551 strain. Plasmid with galactose inducible HO endonuclease pGALHO-pRS412^55^ was a gift from Dr. Jeff Thompson. Strain LS50 with GAL-HO integrated into the genome was a gift from Dr. Lena Ström. This strain was used to create AKY1390. Phosphorylated H2A was detected by anti γ-H2A antibody (ab15083, Abcam). Histone H3 was C-terminally tagged with E2-tag and detected with 5E11 antibody (Icosagen), Rad53 was tagged with Flag-tag and detected with M2 antibody (Sigma-Aldrich). For western blot, cell extracts were prepared as described^56^ and protein samples were separated on SDS-polyacrylamide gel.

### Yeast growth assays and flow cytometry

For growth curve analysis, exponentially growing yeast cultures were inoculated into 10 ml fresh YPD media at density 5 × 10^6^ cells per ml. Cells were grown further in a shaker at 30 °C and samples were collected at indicated time-points during the growth. Culture density was measured with Z2 Cell and Particle Counter (Beckman Coulter). For spot test assays, 10-fold serial dilutions of cell suspensions were made and 5 µl of each dilution was spotted onto plates with synthetic complete (SC) selective medium. For plasmid shuffling, 1 mg/ml 5-fluoroorotic acid (5-FOA) and indicated concentrations of MMS in SC plates were used to test viability of cells. In experiments with Rpb9 anchor-away strains, 1 µg/ml rapamycin (Cayman Europe) in 0.1% DMSO as a final concentration was added to the cultures (0.1% DMSO was used for controls). Plates were incubated at least 2 days at 30 °C. For flow cytometry analysis of cell cycle, 0.5 ml of yeast culture was fixed in 10 ml of ice-cold 70% ethanol for at least 15 min and washed once with 50 mM citric acid. RNA was degraded with RNase A (10 μg/ml) in 50 mM citric acid overnight at 37 °C. DNA was stained with 10× SYBR Green I (Invitrogen) in 50 mM citric acid for 30 min. Cells were analysed with FACS Aria, cell cycle distribution was analysed with Cyflogic software.

### Fluorescence microscopy

For cell morphology analysis, cells were fixed with 70% ethanol and stained with 4′,6-Diamidino-2-Phenylindole (DAPI). Cells were imaged using Olympus BX61 microscope at 100× magnification. Rad52-GFP foci were detected *in vivo* from live S-G2 cells. For quantification at least 100 cells from three independent experiments were counted. For MMS-induction of Rad52 foci, Rpb9 was depleted from cells for 6 hours and treated with 0.1% MMS for 1 hour. All images were collected with cellSens software and analysed in ImageJ.

### DSB repair analysis

For detection of DSB repair in *MAT* locus, cells were grown overnight in rich medium containing 2% raffinose and HO expression was induced for 1.5 hours in the media containing 2% galactose. Then rapamycin was added to the media for depletion of Rpb9 from nucleus and cells were grown for further 1.5 hours in galactose-containing media. After that, HO expression was repressed in 2% glucose-containing media. Cells were collected after each step and at 3, 6 and 20 hour time-points of HO repression in glucose-containing media. Cells were lysed with glass beads and genomic DNA was purified from the cell lysate by phenol-chloroform extraction, followed by chloroform extraction. PCR primers (HOcut-F 5′CTCTGGTAACTTAGGTAAATTACAGC and HOcut-R 5′GAATGATGCTAAGAATTGATTGTTTGC) flanking the HO cut site in *MAT* locus in chromosome III were used to determine the degree of *MAT* locus cutting and repair. *VPS13* locus was amplified with primers VPSq2.6 F (5′ACGTTAATTACTCTTCTGGTTCCG) and VPSq3.5 R (5′AGGTGCCTTGATTACTATGTCCATT) as an internal control. PCR products were resolved in 2% agarose gel electrophoresis, stained with ethidium bromide and quantified with Image Studio Lite.

### Data availability

All data generated or analysed during this study are included in this published article and its Supplementary Information files.

## Electronic supplementary material

Supplementary information
