# Supplementary material for: Rpb9-deficient cells are defective in DNA damage response and require histone H3 acetylation for survival
Source: Sci Rep. 2018 Feb 13;8:2949. doi: 10.1038/s41598-018-21110-9 (PMC5811553; doi:10.1038/s41598-018-21110-9)
Supplement: Supplementary file 1 — Supplementary information [file 41598_2018_21110_MOESM1_ESM.pdf]

Supplementary material for:

Rpb9-deficient cells are defective in DNA damage response and require histone H3 acetylation for survival.

Henel Sein, Kristina Reinmets, Kadri Peil, Kersti Kristjuhan, Signe Värv and Arnold Kristjuhan

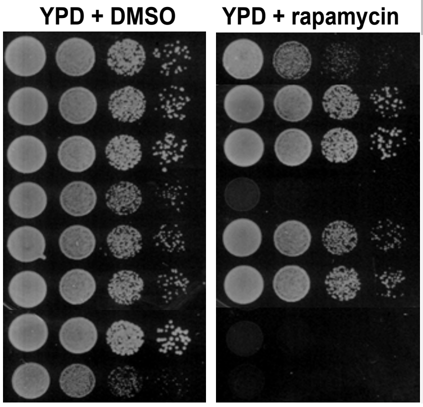

Strain	*RPB9*	RPL13A-2×FKBP12	Histone H3	*TOR1*
AKY1162	*rpb9-FRB*	+	wt	*tor1-1*
AKY1167	*rpb9-FRB*	–	wt	*tor1-1*
AKY1164	*RPB9*	+	wt	*tor1-1*
AKY1190	*rpb9-FRB*	+	H3K9;14;23R	*tor1-1*
AKY1191	*rpb9-FRB*	–	H3K9;14;23R	*tor1-1*
AKY1192	*RPB9*	+	H3K9;14;23R	*tor1-1*
AKY796	*RPB9*	–	wt	*TOR1*
AKY1037	*rpb9∆*	–	wt	*TOR1*

Supplementary Figure S1. Depletion of Rpb9 is lethal in H3 K9,14,23R cells. Anchor-away strains with Rpb9 depletion system along with wild-type strain (AKY796) and *rpb9Δ* mutant (AKY1037) were spotted in 10-fold dilutions onto YPD plates containing DMSO (control) or 1µg/ml rapamycin (for Rpb9 depletion). Plates were photographed after 2 days of incubation. Genetic backgrounds of the strains are summarized in the table. FRB tag (fused to C-terminus of Rpb9 protein) and RPL13A-2xFKBP12 are essential components of the anchor-away system; *tor1-1* mutation is required for bypass from rapamycin-induced cell cycle arrest.

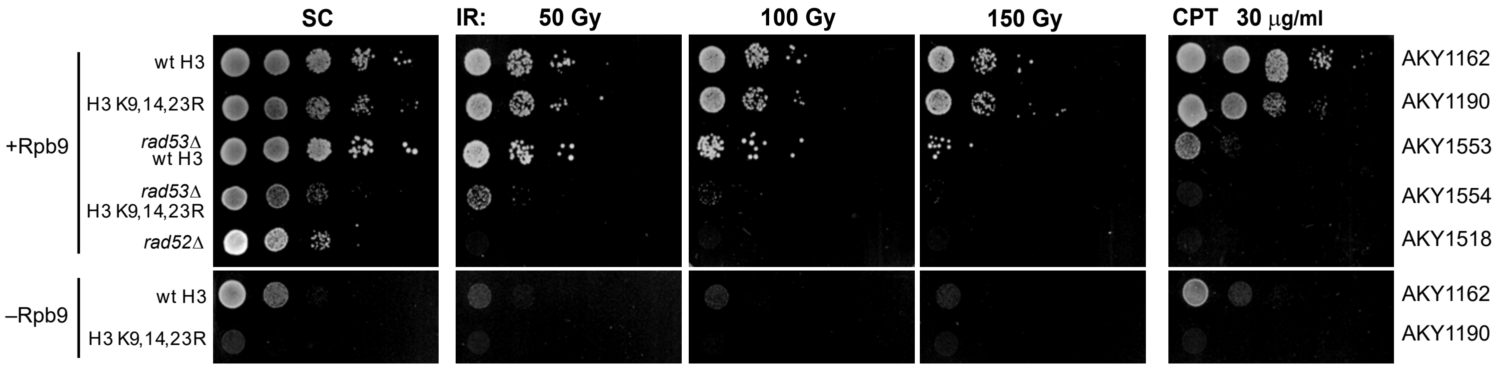

Supplementary Figure S2. 10-fold serial dilutions of Rpb9 anchor-away strains along with *RAD53* and *rad53Δ* strains expressing wt H3 or the H3 K9,14,23R mutant were spotted onto synthetic compleyte (SC) plates containing DMSO (+Rpb9) or rapamycin (–Rpb9). Cells were treated with ionizing radiation (50-150 Gy), or plated on media containing 30 µg/ml camptothecin (CPT). Strains AKY1162 and AKY1190 express rpb9-FRB protein that can be removed from cell nucleus by anchor-away technique. *rad52Δ* strain (AKY1518) is sensitive to various DNA damaging agents and it was used as a positive control for DNA damage induction. Plates were photographed after 3 days of growth at 30°C.

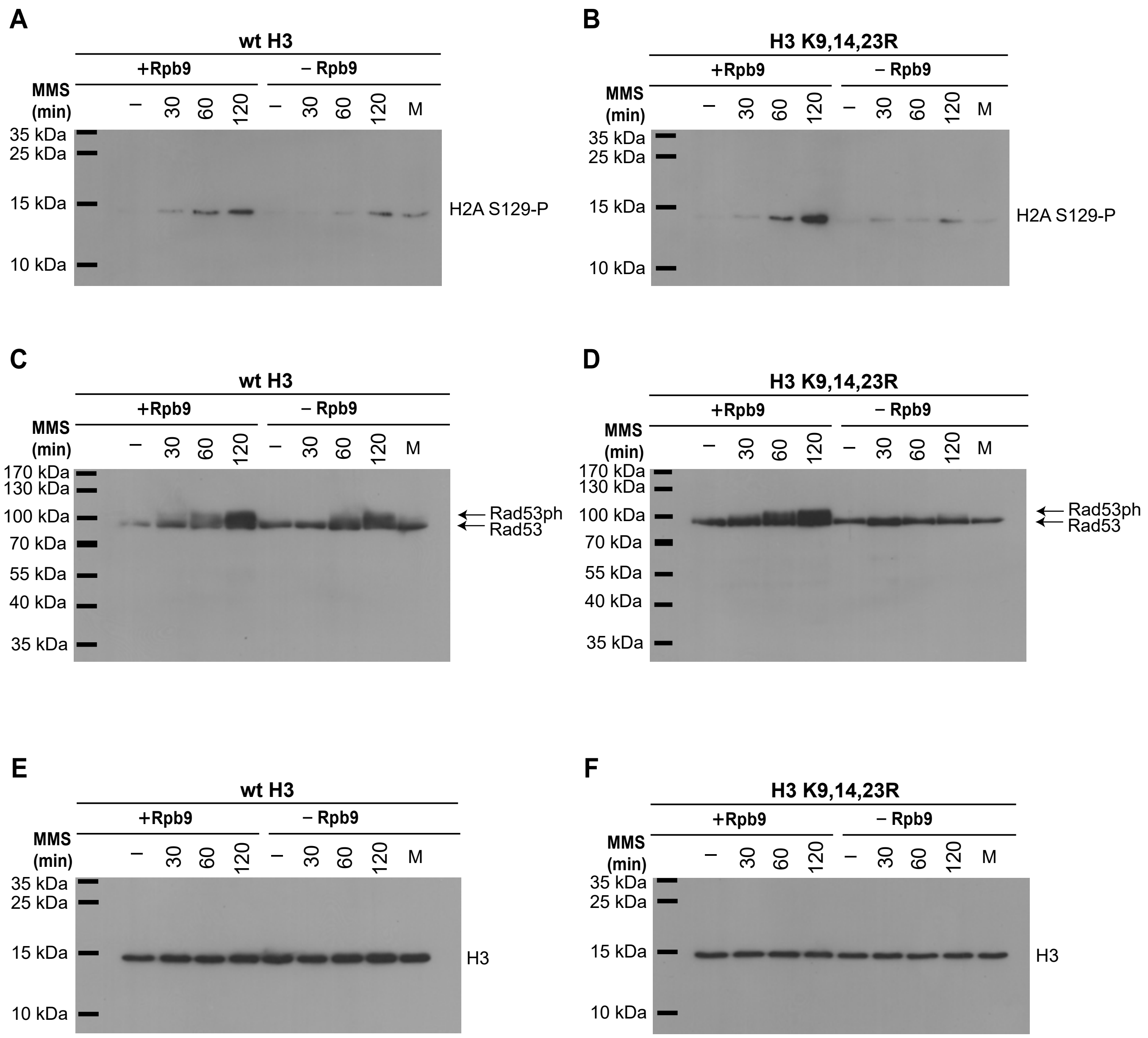

Supplementary Figure S3. Western blot analysis of H2A (**A** and **B**) and Rad53 (**C** and **D**) phosphorylation in response to MMS treatment in Rpb9-depleted cells. Rpb9 anchor-away strains with wt or K9,14,23R mutant H3 were incubated with DMSO (+Rpb9) or rapamycin (–Rpb9) for 6 hours before 0.01% MMS was added to the cells and samples were taken at indicated time-points. Western blot analysis of H3 (**E** and **F**) is shown as a loading control. A positive sample from unrelated experiment was loaded as marker (lane “M” on blots).

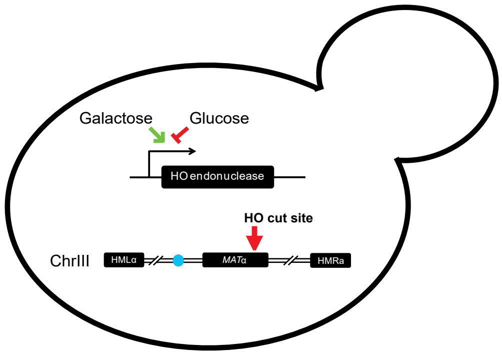

Yeast cells express HO endonuclease under the control of galactose-inducible promoter. In glucose- or raffinose-containing medium, expression of HO endonuclease is repressed. When the cells are shifted to galactose-containing medium, expression of HO is induced and the nuclease makes a DSB at its recognition site in the *MAT* locus.

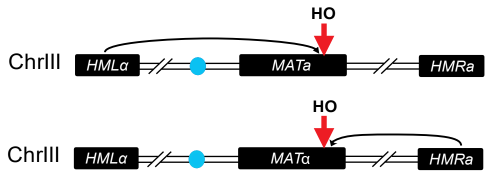

The formed DNA break is repaired primarily by homologous recombination (HR) using the gene cassettes in *HMLα* or *HMRa* loci as donors. As long as the HO endonuclease is expressed, the locus is continuously cut and repaired.

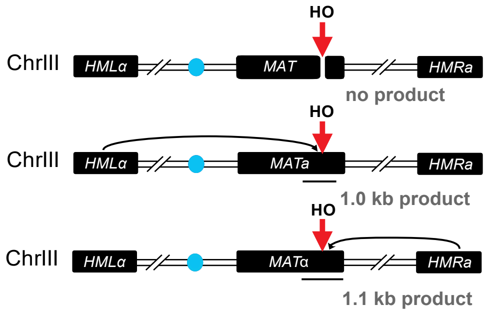

During the course of the experiment, the intactness of the *MAT* locus is detected by PCR. When cells are grown in galactose-containing medium, the majority of cells contain a DSB in HO recognition site and amplification of the *MAT* locus by PCR is strongly reduced. When HO expression is shut-down in glucose-containing medium, the locus is repaired and can be detected by PCR. Depending on the donor sequence used for DSB repair, the lenght of the PCR product can be either 1.0 kb (*MATa*) or 1.1 kb (*MATα*).

Experimental scheme:

1. Raffinose
   ↓
2. Galactose (1.5 h)
   ↓
3. Galactose + rapamycin (or DMSO) (1.5 h)
   ↓
4. Glucose + rapamycin (or DMSO) (3h, 6h, 20h)

1. Cells are pre-grown in raffinose-containing medium. Expression of HO endonuclease is OFF.

2. Expression of HO nuclease is induced in galactose-containing medium for 1.5 hours to induce DSBs in the *MAT* locus.

3. Rapamycin (or DMSO) is added to growth medium to induce depletion of Rpb9 protein from the cell nucleus. Cells are grown for further 1.5 hours. Expression of HO endonuclease is ON.

4. Expression of HO endonuclease is shut-off by transferring the cells into glucose-containing medium. DNA samples are collected at different time-points to estimate the efficiency of *MAT* locus repair in the presence or absence of Rpb9 protein in the cell nucleus (DMSO and rapamycin samples, respectively).

Supplementary Figure S4. Overview of the DSB repair assay. In all strains the galactose-inducible HO endonuclease is integrated into *ade3* locus and Rpb9 protein can be removed from cell nucleus by the anchor-away technique. HO cuts its recognition site in the *MAT* locus in chromosome III. The site is repaired mainly by homologous recombination using the *HMLα* or *HMRa* loci in the same chromosome as donor sequences. Location of the chromosome III centromere relative to the other loci is indicated as a blue circle. Red arrow above the *MAT* locus indicates the HO cut site and black bars under the *MAT* locus indicate the region amplified by PCR to estimate the intactness of the locus.

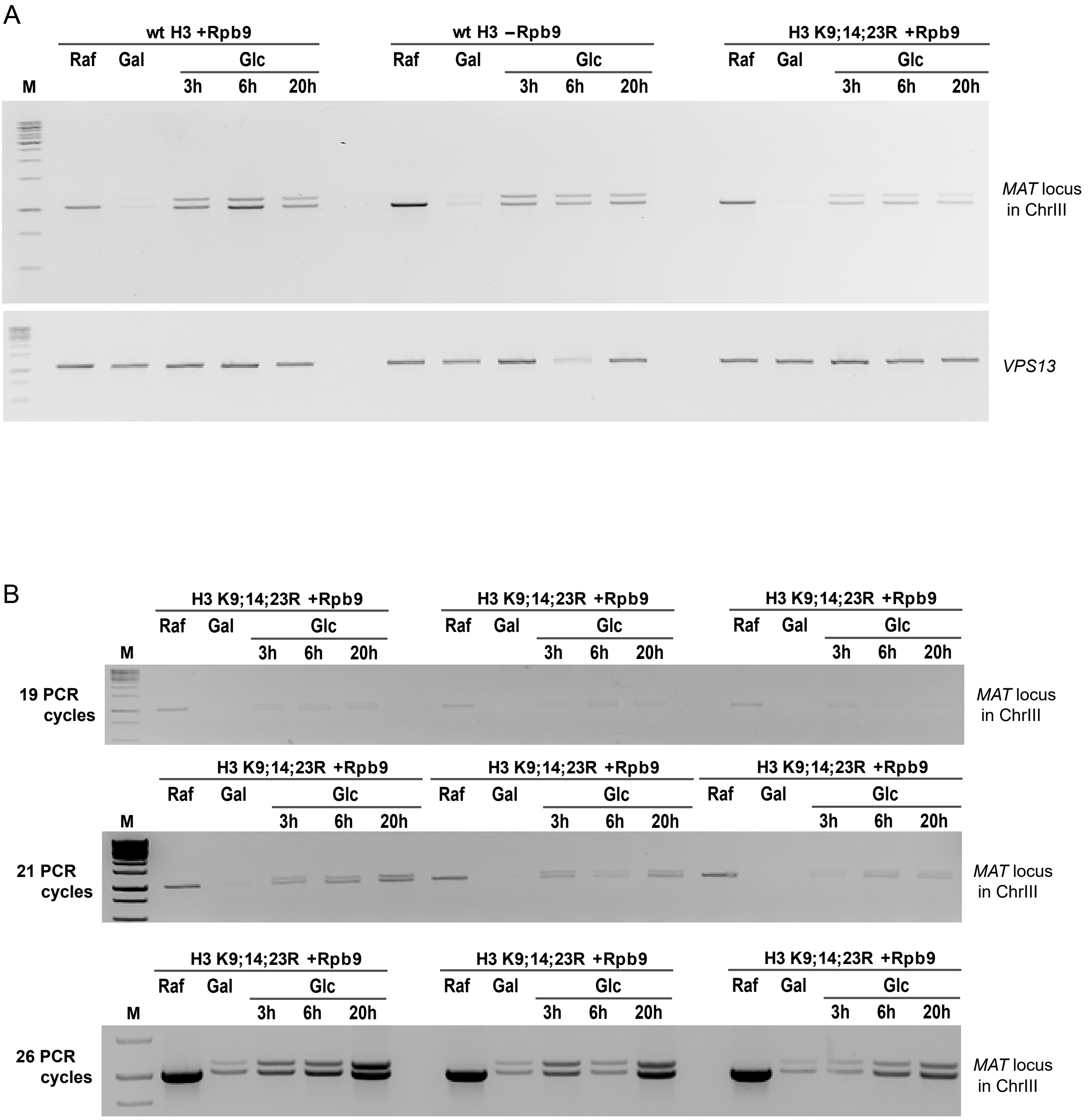

Supplementary Figure S5. (A and B) Agarose gel electrophoresis analysis of HO cutting and repair. PCR products were obtained from cells before HO induction (Raf), 3 hours after HO induction (Gal) and 3, 6 or 20 hours after repression of HO (Glc). PCR product of the *VPS13* locus was used as an internal control. Thermo Scientific GeneRuler 1 kb DNA ladder (M) was used. **(B)** Different PCR cycles (19, 21, 26) were tested to make sure that the PCR product formation was in linear range.

Supplementary Table S1. Yeast strains

Strain	Genotype	Source
AKY796	*W303, MAT α, hht1-hhf1::LEU2 gal-pr-vps13::TRP1 hht2-hhf2::kanMX RPB9-natMX6 Ycp50:hht2-hhf2 (URA3)*	This study
AKY1037	*MAT α, hht1-hhf1::LEU2 gal-pr-vps13::TRP1 hht2-hhf2::kanMX rpb9::natMX6 YCp50:hht2-hhf2 (URA3)*	This study
AKY1116	MAT α, *rpl13a-2-fkbp12::TRP1 fpr1::natMX6 tor1-1 rpb9-frb::hghMX*	This study
AKY1162	*MAT A, rpl13a-2-fkbp12::TRP1 fpr1::natMX6 tor1-1 rpb9-frb::hghMX hht1-hhf1::LEU2 hht2-hhf2::kanMX [pRS413-H3H4-3F12-wt H3(HIS3)]*	This study
AKY1164	*MAT α, rpl13a-2-fkbp12::TRP1 fpr1::natMX6 tor1-1 hht1-hhf1::LEU2 hht2-hhf2::kanMX [pRS413-H3H4-3F12-wt H3(HIS3)]*	This study
AKY1167	*MAT A, fpr1::natMX6 tor1-1 rpb9-frb::hghMX hht1-hhf1::LEU2 hht2-hhf2::kanMX [pRS413-H3H4-3F12-wt H3(HIS3)]*	This study
AKY1190	*MAT A, rpl13a-2-fkbp12::TRP1 fpr1::natMX6 tor1-1 rpb9-frb::hghMX hht1-hhf1::LEU2 hht2-hhf2::kanMX [pRS413-H3H4-3F12-H3 K9,14,23R(HIS3)]*	This study
AKY1191	*MAT A, fpr1::natMX6 tor1-1 rpb9-frb::hghMX hht1-hhf1::LEU2 hht2-hhf2::kanMX [pRS413-H3H4-3F12-H3 K9,14,23R(HIS3)]*	This study
AKY1192	*MAT α, rpl13a-2-fkbp12::TRP1 fpr1::natMX6 tor1-1 hht1-hhf1::LEU2 hht2-hhf2::kanMX[pRS413-H3H4-3F12-H3 K9,14,23R(HIS3)]*	This study
AKY1391	*MAT α, rpl13a-2-fkbp12::TRP1 fpr1::natMX6 tor1-1 rpb9-frb::hghMX hht1-hhf1::LEU2 hht2-hhf2::kanMX [pRS413-H3H4-3F12- wt H3(HIS3)] GAL-HO::ade3*	This study
AKY1399	*MAT α, rpl13a-2-fkbp12::TRP1 fpr1::natMX6 tor1-1 rpb9-frb::hghMX hht1-hhf1::LEU2 hht2-hhf2::kanMX [pRS413-H3H4-3F12- H3 K9,14,23R(HIS3)] GAL-HO::ade3*	This study
AKY1508	*MAT α, gal-pr-vps13::TRP1 hht1-hhf1::LEU2 hht2-hhf2::kanMX YCp50:hht2-hhf2 (URA3) sml1::hghMX*	This study
AKY1509	*MAT α, gal-pr-vps13::TRP1 hht1-hhf1::LEU2 hht2-hhf2::kanMX YCp50:hht2-hhf2 (URA3) sml1::hghMX rad53::natMX6*	This study
AKY1518	*MAT A, GAL-HO::ade3 rad52::natMX6*	This study
AKY1551	*MAT A, ADE2, rpl13a-2-fkbp12::TRP1 fpr1::natMX6 tor1-1 rpb9-frb::hghMX hht1-hhf1::LEU2 hht2-hhf2::kanMX [pRS413-H3H4-3F12-wt H3(HIS3)] rad52-yEGFP::URA3*	This study
AKY1553	*MAT α, gal-pr-vps13::TRP1 hht1-hhf1::LEU2 hht2-hhf2::kanMX [pRS413-H3H4-3F12-wt H3(HIS3)] sml1::kanMX rad53::URA3*	This study
AKY1554	*MAT α, gal-pr-vps13::TRP1 hht1-hhf1::LEU2 hht2-hhf2::kanMX [pRS413-H3H4-3F12-H3 K9,14,23R(HIS3)] sml1::kanMX rad53::URA3*	This study
AKY1564	*MAT A, ADE2, rpl13a-2-fkbp12::TRP1 fpr1::natMX6 tor1-1 rpb9-frb::hghMX hht1-hhf1::LEU2 hht2-hhf2::kanMX [pRS413-H3H4-3F12-H3 K9,14,23R(HIS3)] rad52-yEGFP::URA3*	This study
AKY1588	*MAT A, rpl13a-2-fkbp12::TRP1 fpr1::natMX6 tor1-1 rpb9-frb::hghMX hht1-hhf1::LEU2 hht2-hhf2::kanMX [pRS413-H3H4-3F12-wt H3(HIS3)] rad53-3xFlag::URA3*	This study
AKY1589	*MAT A, rpl13a-2-fkbp12::TRP1 fpr1::natMX6 tor1-1 rpb9-frb::hghMX hht1-hhf1::LEU2 hht2-hhf2::kanMX [pRS413-H3H4-3F12-H3 K9,114,23R(HIS3)] rad53-3xFlag::URA3*	This study

Supplementary Table S2. Yeast plasmids

Plasmid	Description	Source
pRS413-H3H4-3F12	CEN, *HIS3*, H3-3F12; H4	This Study
pRS413-H3H4-3F12-H3K9R	CEN, *HIS3*, H3K9R-3F12; H4	This Study
pRS413-H3H4-3F12-H3K14R	CEN, *HIS3*, H3K14R-3F12; H4	This Study
pRS413-H3H4-3F12-H3K18R	CEN, *HIS3*, H3K18R-3F12; H4	This Study
pRS413-H3H4-3F12-H3K23R	CEN, *HIS3*, H3K23R-3F12; H4	This Study
pRS413-H3H4-3F12-H3K27R	CEN, *HIS3*, H3K27R-3F12; H4	This Study
pRS413-H3H4-3F12-H3K9;14R	CEN, *HIS3*, H3K9;14R-3F12; H4	This Study
pRS413-H3H4-3F12-H3K9;18R	CEN, *HIS3*, H3K9;18R-3F12; H4	This Study
pRS413-H3H4-3F12-H3K9;23R	CEN, *HIS3*, H3K9;23R-3F12; H4	This Study
pRS413-H3H4-3F12-H3K9;27R	CEN, *HIS3*, H3K9;27R-3F12; H4	This Study
pRS413-H3H4-3F12-H3K14;18R	CEN, *HIS3*, H3K14;18R-3F12; H4	This Study
pRS413-H3H4-3F12-H3K14;23R	CEN, *HIS3*, H3K14;23R-3F12; H4	This Study
pRS413-H3H4-3F12-H3K14;27R	CEN, *HIS3*, H3K14;27R-3F12; H4	This Study
pRS413-H3H4-3F12-H3K18;23R	CEN, *HIS3*, H3K18;23R-3F12; H4	This Study
pRS413-H3H4-3F12-H3K18;27R	CEN, *HIS3*, H3K18;27R-3F12; H4	This Study
pRS413-H3H4-3F12-H3K23;27R	CEN, *HIS3*, H3K23;27R-3F12; H4	This Study
pRS413-H3H4-3F12-H3K9;14;18R	CEN, *HIS3*, H3K9;14;18R-3F12; H4	This Study
pRS413-H3H4-3F12-H3K9;14;23R	CEN, *HIS3*, H3K9;14;23R-3F12; H4	This Study
pRS413-H3H4-3F12-H3K9;14;27R	CEN, *HIS3*, H3K9;14;27R-3F12; H4	This Study
pRS413-H3H4-3F12-H3K9;18;23R	CEN, *HIS3*, H3K9;18;23R-3F12; H4	This Study
pRS413-H3H4-3F12-H3K9;18;27R	CEN, *HIS3*, H3K9;18;27R-3F12; H4	This Study
pRS413-H3H4-3F12-H3K9;23;27R	CEN, *HIS3*, H3K9;23;27R-3F12; H4	This Study
pRS413-H3H4-3F12-H3K14;18;23R	CEN, *HIS3*, H3K14;18;23R-3F12; H4	This Study
pRS413-H3H4-3F12-H3K14;18;27R	CEN, *HIS3*, H3K14;18;27R-3F12; H4	This Study
pRS413-H3H4-3F12-H3K14;23;27R	CEN, *HIS3*, H3K14;23;27R-3F12; H4	This Study
pRS413-H3H4-3F12-H3K18;23;27R	CEN, *HIS3*, H3K18;23;27R-3F12; H4	This Study
pRS413-H3H4-3F12-H3K9;14;18;23R	CEN, *HIS3*, H3K9;14;18;23R-3F12; H4	This Study
pRS413-H3H4-3F12-H3K9;14;18;27R	CEN, *HIS3*, H3K9;14;18;27R-3F12; H4	This Study
pRS413-H3H4-3F12-H3K9;14;23;27R	CEN, *HIS3*, H3K9;14;23;27R-3F12; H4	This Study
pRS413-H3H4-3F12-H3K9;18;23;27R	CEN, *HIS3*, H3K9;18;23;27R-3F12; H4	This Study
pRS413-H3H4-3F12-H3K14;18;23;27R	CEN, *HIS3*, H3K14;18;23;27R-3F12; H4	This Study
pRS413-H3H4-3F12-H3K9;14;18;23;27R	CEN, *HIS3*, H3K9;14;18;23;27R-3F12; H4	This Study
